# Supplementary material for: Efficient parameter calibration and real-time simulation of large-scale spiking neural networks with GeNN and NEST
Source: Front Neuroinform. 2023 Feb 10;17:941696. doi: 10.3389/fninf.2023.941696 (PMC9950635; doi:10.3389/fninf.2023.941696)
Supplement: Supplementary file 1 [file Data_Sheet_1.pdf]

# Supplementary Material

## 1 - Supplemental figures

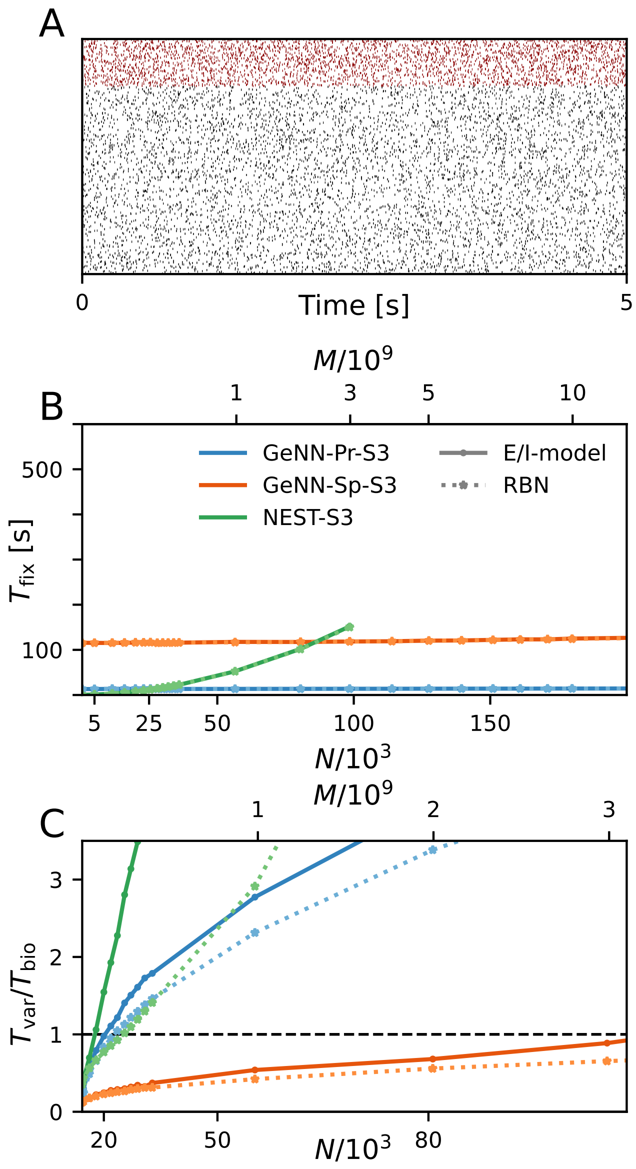


Figure S1: **Spiking activity and costs of simulation for the random balanced network (RBN).** (A) Spike raster plot of excitatory (black) and inhibitory (red) neurons in a network of $N = 25,000$ neurons during 5 s of spontaneous activity. The network activity does not show metastable behavior. Shown are 8% of the total neuron population. Network parameters are $J_{E+} = 1.0$, $I_{thE} = 2.6$, $I_{thI} = 1.9$. The spike raster plot was generated from a GeNN simulation. (B) The fixed costs of simulation are indistinguishable for the E/I model with homogeneous synaptic time constants (solid lines) and the RBN model (dotted lines) for both GeNN (blue and orange lines) and NEST (green lines). (C) NEST and GeNN have decreased variable costs for the RBN model.

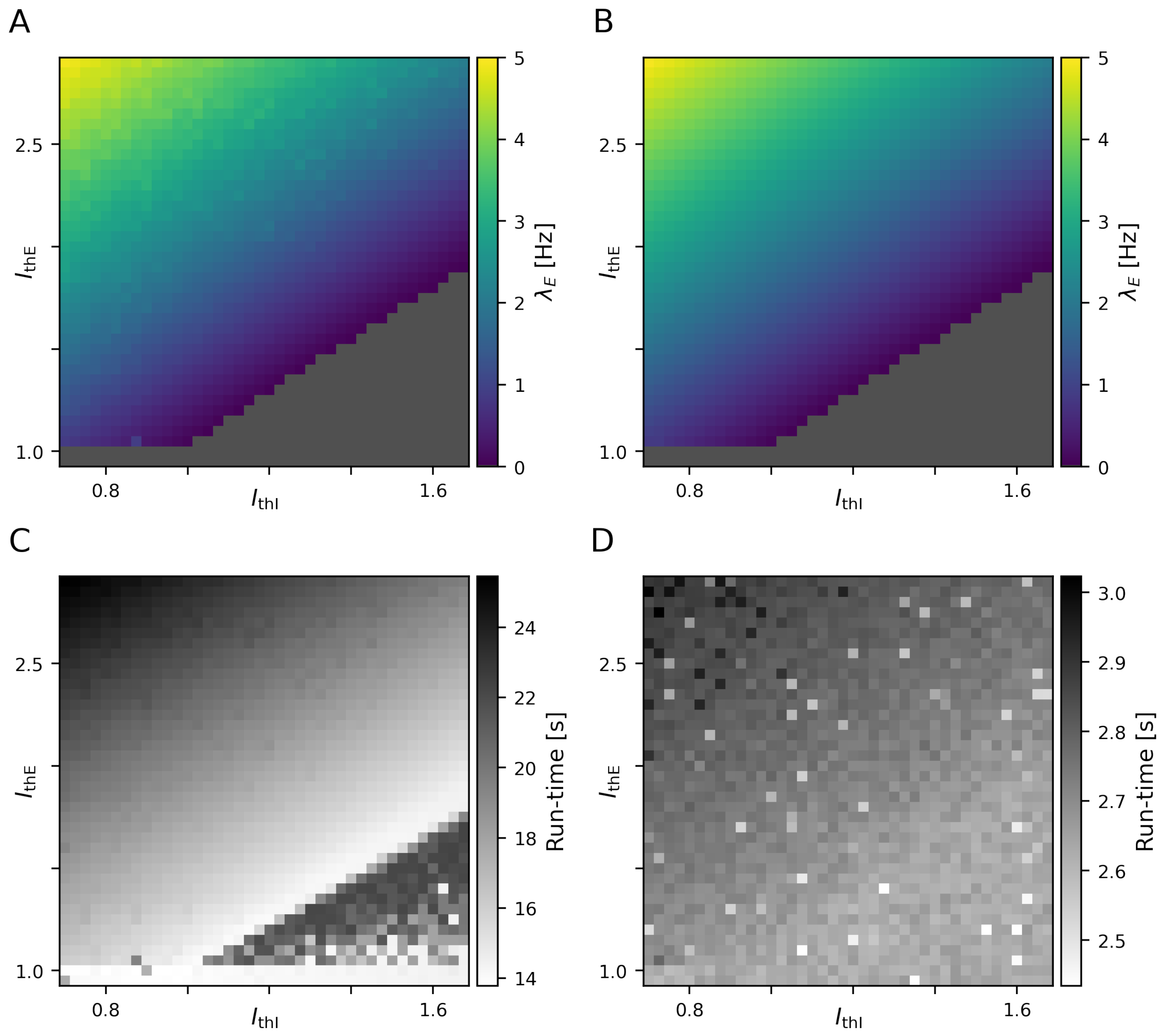


Figure S2: **Run-time dependence on average firing rates during grid search.** (A, B) Average spontaneous firing rates of all excitatory neurons in dependence on the constant input currents provided to all excitatory ($I_{\mathrm{thE}}$) and inhibitory ($I_{\mathrm{thI}}$) neurons in a network of $N = 25,000$ neurons (cf. Fig. 5 B and C in the main text). Simulations with NEST were run sequentially on S3 with 24 threads (A) and with a batch size of one in GeNN (B). All simulations comprise 10 seconds of biological model time. (C) The wall-clock time of simulations with NEST shows a strong dependence on the average firing rate per network. In the lower right of the matrix, the simulation time was increased due to the activity of inhibitory neurons (not shown in A,B). (D) Wall-clock time of simulations with GeNN. The reported times include reinitialization of the model, simulation of the network, transfer and processing of the spikes and storing of the result. In addition to these costs, the complete grid search required 117 s to build, compile and load the model (additional 0.07 s for each simulation). The run-time of GeNN simulations shows only a weak dependence on the average firing rate.

## 2 - Practical guide to GeNN implementation

In the following, we provide technical details on setting up different parameters that helped us to implement our model successfully in GeNN.

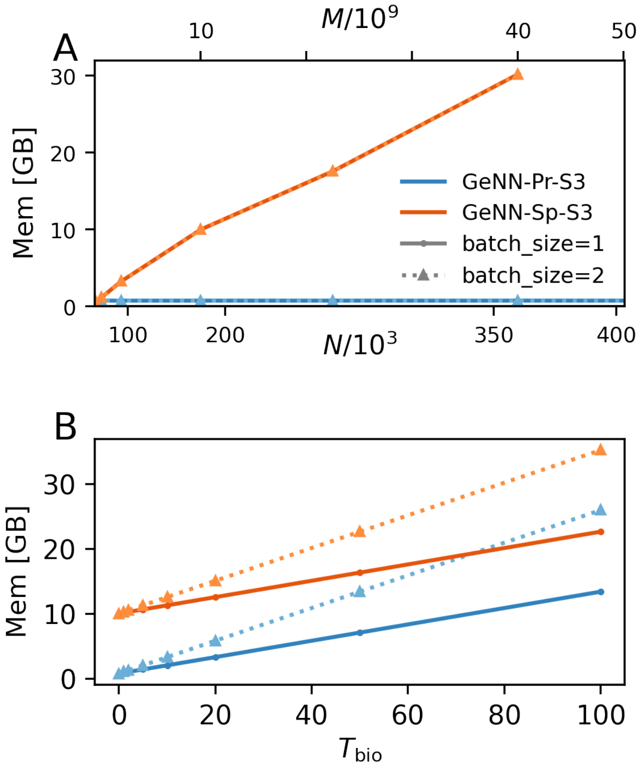


Figure S3: **Memory consumption on the GPU.** (A, B) Total allocated GPU memory on NVIDIA RTX A6000 (S3). Memory usage was measured with the NVIDIA Management Library by using Python bindings (nvidia-ml-py3) without any other processes on the GPU. The memory consumption is measured after the network model is loaded, including a spike recorder that is large enough to fit all time steps to be simulated. The partitioning scheme (see Materials and Methods) is deactivated for these simulations. (A) Network models are loaded with a spike recorder of size zero. Thus, the spike recorder is non-existent and spikes are only present in the buffers of the simulation. The simulation without a spike recorder allows the quantification of the memory consumption of the model independent of simulation duration. Memory consumption in dependence on network size is independent of the batch size (solid lines versus dotted lines). Memory consumption stays constant when using the PROCEDURAL approach (GeNN-Pr-S3) over the whole range of simulated network sizes, but grows monotonically and approximate linearly with the number of synapses when using the SPARSE approach (GeNN-Sp-S3). (B) For a network with 100,000 neurons, total memory consumption increases monotonically with the size of the spike recorder that grows linearly with the number of time steps that are simulated. The number of batches multiplies the memory space to be allocated to the spike recorder and thus a simulation with a batch size of 2 consumes more memory than a simulation with a batch size of 1.

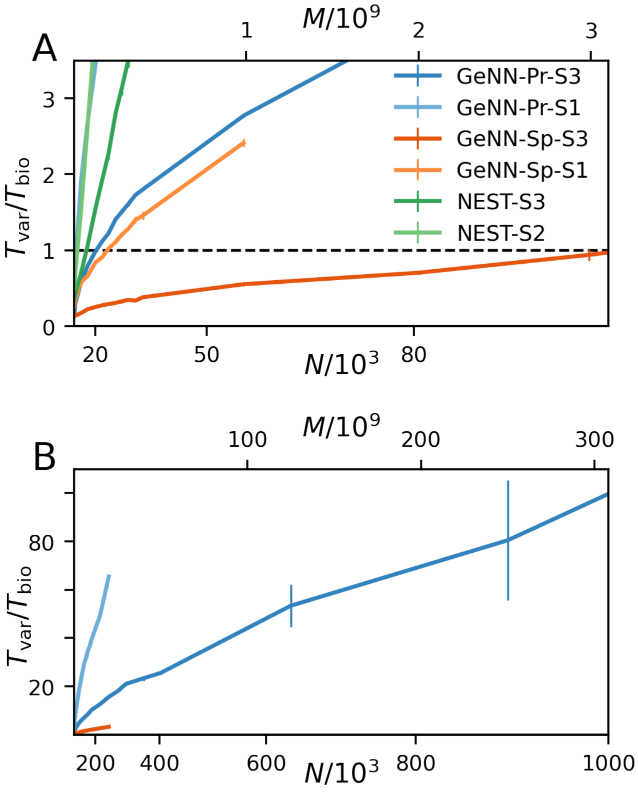


Figure S4: **Variable costs of simulation and real-time limitation.** (A, B) Mean and standard deviation of the variable cost factor $T_{\mathrm{var}}/T_{\mathrm{bio}}$ across 10 repeated simulations for each data point. The x-axis is linear in M (top). (A) The standard deviation is small for all hardware configurations and both simulators in the displayed range of network sizes. The curves for GeNN-Pr-S1 and NEST-S2 are almost congruent. (B) The standard deviation grows with network size and for very large networks that could be simulated only with the PROCEDURAL approach (GeNN-Pr-S3). This figure compares to Fig. 3 in the main text, where the median across 10 simulations is shown.

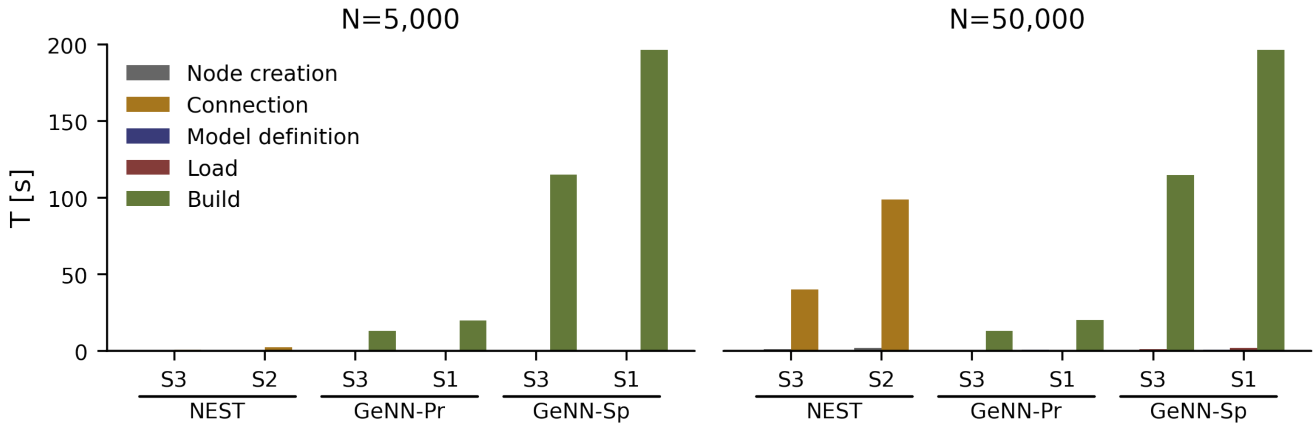


Figure S5: **Fixed costs of simulation.** Individual costs for execution phases of NEST and GeNN for two network sizes of $N = 5,000$ and $N = 50,000$ neurons. The GPU-based simulation requires expensive model compilation (Build). NEST uses pre-compiled neuron models. Same data as in Fig. 2 A

**Extra_global_parameters** We used extra global parameters to avoid recompilation of the model during the grid search. Global parameters can be set as scalar or as pointer and have to be initialized before the model is loaded. We choose in all cases pointers to prevent GeNN from copying the value of the global parameter at every simulation step between host and GPU. This speeds up the simulation, but needs the manual transfer of the data to the GPU after global parameters are changed and the model is loaded into the GPU.

**Variable locations** GeNN allows the setting of the locations of variables, which include state variables of the model and connectivity matrices. GeNN allocates memory on the host and on the GPU in its standard settings. This allows the pulling of variables from the GPU to the host during the simulation. Most of the memory on the host is never used during the simulation if the specific variable is never pulled. The default locations of variables can be changed by setting `model.default_var_location` for the location of variables or `model.default_sparse_connectivity_location` for the location of the sparse connectivity matrix to either `genn_wrapper.VarLocation_DEVICE` or `genn_wrapper.VarLocation_HOST_DEVICE` to allocate memory only on the GPU or on the host device and the GPU respectively. Locations of single variables can be changed by `GROUPNAME.set_var_location(VARNAME, LOCATION)`. Similar commands exist for the location of spikes and spikelike events.

**Connectivity matrices** GeNN allows the pulling of sparse connectivity matrices. This requires that the location of the connectivity matrix is set to both GPU and host. Connectivity is pulled with the command `SynapseGroup.pull_connectivity()`. Afterwards the connectivity can be accessed by using the commands `SynapseGroup.get_sparse_pre_inds()`, `SynapseGroup.get_sparse_post_inds()` and `SynapseGroup.get_var_values(..)`. All three lists are in the same order. Indices of pre- and postsynaptic neurons are specific to the `SynapseGroup`.

**Code generation** GeNN models can grow very large and can lead to problems during the compilation of the code. The setting of the locations of variables to only GPU leads to a reduction in number of variables, thus to a reduction of code size. This may enable the compiler to compile larger models and be faster at the cost of a missing pull command for these variables.
On the same track, the setting of the options `generateEmptyStatePushPull=False` and `generateExtraGlobalParamPull=False` for the `model.build()` reduces the code size with the cost of not implementing a pull function for the global parameters. We have chosen to always set these options to false. During the testing of our model, we had to set the variable location to only GPU to be able to compile models with a larger number of clusters. With 20 clusters, this is not needed.

**Random seed** The random seed of a model can be set with `model._model.set_seed(SEED)`. The seed applies to all model instances in a batch.

**Batch size** As soon as the CUDA-cores of the GPU are fully utilized during each simulation step an increase in the `batch_size` does not lead to significantly speed up of the whole simulation.

**GPU cooling** GeNN has high demands on the GPU in terms of its power consumption. To our experience, with insufficient cooling, the GPU will heat up fast and throttle its frequency. This leads to a reduction in simulation speed. The fan-curves of NVIDIA GPUs are typically balanced between the noise level and the cooling performance. If the noise level is irrelevant, an increase of the fan speed to its maximum value before a simulation with GeNN might lead to slightly faster simulations.

**Information resources** The GeNN documentation offers information and guidance for setting up a GeNN model. We found it additionally helpful to look into the following files of GeNN to look up functions and usages which were quite specific to our use case of the grid search. `genn/pygenn/genn_model.py`

includes all functions for the creation of custom models and the basic class for the GeNN model. `genn/pygenn/genn_groups.py` includes all functions for the manipulation of neuron- and synapse groups. This includes the setting of global parameters as well the pulling operations for the connectivity and state variables. For the implementation of our tunable parameters of neuron models, post-synaptic dynamics and connectivity initialization snippets, we looked mainly into the header files in `genn/include/genn/genn/` like `postsynapticModels.h`. The implementations there are directly transferable into custom implementations in PyGeNN.
Due to the declaration as strings of the main part of the custom models, we found it hard to find missing brackets or delimiters in these lines. We found it very helpful to look into the generated c-code like neuronUpdate.cc. During the compilation of the generated code, the compiler will output the respective file and in brackets the line number of an error. As this is plain c++ code, typical editors will highlight the code and color missing brackets or semicolons. The generated code in these lines is easily relatable to the model definitions.

## Example implementations

In this section we want to provide examples how to tune different parameters which occur in most SNNs in GeNN without the need of recompilation. GeNN models are modular and based on self-definable models. These are for example neuron models, current sources, synapse models which consists of different submodels and variable initializations like the connectivity or any state variable in the model which are freely combinable. Most of these self-definable models allow so-called `extra_global_params`. These parameters are variables which are alterable by the user during runtime. This allows for example to generate a reward signal for synapses at every simulation time-step or the change of model parameters without recompilation of the model. An example of such a model and its usage in a GeNN model with creation and setting the parameter is presented in the following listing.

```
# Model definition
def define_stimulus():
   stimulus = genn_model.create_custom_current_source_class("stim",
   extra_global_params=[("Ix", "float*")],
   injection_code="""
      $(injectCurrent, + $(Ix)[$(batch)]);
   """)
return stimulus

# Creation in GeNN model
stimulus_model = define_stimulus()
Stimulus = model.add_current_source(
   "Stimulus", stimulus_model, target_neuron_group, {}, {})

# Setting parameter before model_load
Stimulus.set_extra_global_param("Ix", [value,...])

# Setting parameter after loading
Stimulus.extra_global_params["Ix"].view[:] = [value,...]
Stimulus.push_extra_global_param_to_device("Ix", size)
```

The supplied code is currently specific to a GPU as backend. The variable `batch` allows for different parameters in instances of a batch. This variable is not set for the CPU backend in the GeNN version 4.7. This will change in upcoming versions and allow for the application of the same model on all backends. Another important difference between the GPU backend and the CPU backend is the dual memory on the GPU backend. We use pointers for the variables, as they lead to a more lightweight and faster code. But

due to the divided memory, we have to manually push any changes to a parameter after the model is loaded into the GPU. This is not necessary on the CPU backend.

In the following we show examples of implementations, which utilize different model classes of GeNN. Other tunable parameters can be implemented similarly. We only provide here the definition of the global parameter and the relevant line of the simulation code of the model definition and its usage and setting in the actual GeNN model. The complete implementation details of the models can be found in the GitHub repository.

**Direct current stimulation** Our clustered model needs a background stimulation to produce neural activity. This can be implemented with a current source as shown in the example above or by an implementation in the neuron model.

```
# Model definition
neuron_model=genn_model.create_custom_neuron_class("neuron_model"
extra_global_params=[("Ix", "float*")],
sim_code="""
    ...
    $(V) = $(P30) * ($(Ioffset) + $(Isyn)+ $(Ix)[$(batch)]) + ...
    ...
"""
return neuron_model

# Creation in GeNN model
Neuron_group=model.add_neuron_population(NAME_NG, SIZE_NG,
             neuron_model, NEURON_PARAMS, NEURON_INIT)

# Setting parameter before model_load
Neuron_group.set_extra_global_param("Ix", [value,...])

# Setting parameter after loading
Neuron_group.extra_global_params["Ix"].view[:] = [value,...]
Neuron_group.push_extra_global_param_to_device("Ix", size)
```

**Membrane time constant** A tunable membrane time constant $\tau_m$ needs some modifications to our chosen integration scheme. The exact integration scheme leads to the factor $exp(-\Delta t/\tau_m)$ in all propagators of the neuron model. The calculation of the exponential function is computationally demanding and there is no advantage in calculating it in the simulation code at every simulation step. We decided to supply this factor as a global parameter to calculate the decay of the membrane potential and to switch for the propagation of the input to the Euler scheme, as it would also need the time constant itself. If more parameters should be tunable, it may be advisable to calculate all propagators as global parameters.

```
# Model definition
neuron_model=genn_model.create_custom_neuron_class("neuron_model"
extra_global_params=[("PropTauM", "float*")],
sim_code="""
    ...
    $(V) = $(PropTauM)[$(batch)] * $(V) + (1-$(PropTauM)[$(batch)])
                  * $(Vrest) + DT*($(Ioffset) + $(Isyn))/$(C);
    ...
"""
return neuron_model

# Creation in GeNN model
Neuron_group=model.add_neuron_population(NAME_NG, SIZE_NG,
             neuron_model, NEURON_PARAMS, NEURON_INIT)
```

```
# Setting parameter before model_load
Neuron_group.set_extra_global_param("PropTauM",
                                 [np.exp(-dt/tau_value),...])

# Setting parameter after loading
Neuron_group.extra_global_params["PropTauM"].view[:] =
                                  [np.exp(-dt/tau_value),...]
Neuron_group.push_extra_global_param_to_device("PropTauM", size)
```

**Synapse weights** We implemented tunable synapse weights in the synapse groups and not in the neuron models. This ensures modularity.

```
# Model definition
WeightSyn = genn_model.create_custom_weight_update_class("WeightSyn",
extra_global_params=[("g", "float*")],
sim_code="""
   $(addToInSyn, $(g)[$(batch)]);
"""
return WeightSyn

# Creation in GeNN model
Synapse_group=model.add_synapse_population(NAME_SG,
                MATRIX_TYPE, delaySteps,
                SOURCE_NG, TARGET_NG,
                WeightSyn, {}, {}, {}, {},
                Postsyn_Dyn, PostSyn_PARAMS, {},
                CONNECTIVITY_INIT)

# Setting parameter before model_load
Synapse_group.set_extra_global_param("g", [value,...])

# Setting parameter after loading
Synapse_group.extra_global_params["g"].view[:] = [value,...]
Synapse_group.push_extra_global_param_to_device("g", size)
```

**Synapse time constant** We did not implement a tunable synapse time constant. In its current version 4.7, GeNN does not implement the `extra_global_params` in the postsynaptic class, which would be needed to implement it modularly. This should be changed in an upcoming version and can be done similar to the other examples. To ensure speed during simulation, the global parameter should be the decay factor $exp(-\Delta t/\tau_{synapse})$ instead of the time constant itself. If the tuning of synaptic time constants is needed for only a few synapse types, the decay dynamics can be implemented in the neuron model by adding synaptic input as Dirac impulse to an additional state variable. But this is not advisable as it has to be done for every neuron model and is not scaleable as for every synapse type one decay dynamic has to be implemented in the neuron model.

**Connectivity density** GeNN uses one set of connectivity matrices (each synapse group has one) for all model instances in a batch. These matrices are initialized during the loading of the model or by calling `model.reinitalize`. They are not alterable during a simulation. A grid search has to be designed in a way that it ensures same connectivity densities in a batch. In our model, we had to use two different kinds of connectivity initializations: `FixedProbability` and `FixedProbabilityNoAutapse`. We had to replicate both classes to make the connectivity density of our model tunable. The custom class should provide apart from the initialization itself functions to calculate the maximum row length and the maximum column length of the connectivity matrix. These functions are needed during the optimization of the CUDA code, thus the maximum size is fixed as long as the model is not recompiled.

If the number of connections is actually bigger as the results of the functions provided, the results will be wrong. If a size is chosen which is much bigger than needed, more memory in the GPU and dependent on the settings also in the RAM will be used. This may reduce the maximum size of a network or the maximum `batch_size`. Fewer entries in the matrices are handled by GeNN. The calculation of the value $1/(log(1-p))$ follows the same rational as the calculation of the exponential function outside the simulation code. The `row_build_code` is executed during the initialization of the connectivity matrix for each row one time and the calculation of the logarithm would consume computational power without having any advantage.

```
# Model definition
ConnDensity = genn_model.
   create_custom_sparse_connect_init_snippet_class('Conn_Density',
extra_global_params=[("pp", "float*")],
row_build_state_vars=[("prevJ", "int", -1)],
row_build_code="""
   const scalar u = $(gennrand_uniform);
   prevJ += (1 + (int)(log(u) * $(pp)[0]));
   if(prevJ < $(num_post)) {
      $(addSynapse, prevJ + $(id_post_begin));
   }
   else {
      $(endRow);
   }
""",
calc_max_row_len_func=genn_model.create_cmlf_class(
         lambda num_pre, num_post, pars: int(num_post))(),
calc_max_col_len_func=genn_model.create_cmlf_class(
         lambda num_pre, num_post, pars: int(num_pre))())
return ConnDensity

# Creation in GeNN model
CONNECTIVITY_INIT= genn_model.init_connectivity(
                    ConnDensity,{})
Synapse_group=model.add_synapse_population(NAME_SG,
                           MATRIX_TYPE, delaySteps,
                           SOURCE_NG, TARGET_NG,
                           "StaticPulse", {}, WEIGHT_PARAM, {}, {},
                           Postsyn_Dyn, PostSyn_PARAMS, {},
                           CONNECTIVITY_INIT)

# Setting parameter before model_load
Synapse_group.connectivity_extra_global_params['pp'].
         set_values([1.0/np.log(1.0-Probability)])

# Setting parameter after loading
Synapse_group.connectivity_extra_global_params["pp"].view[:] =
                            [1.0/np.log(1.0-Probability)]
Synapse_group._push_extra_global_param_to_device("pp",
            egp_dict=Synapse_group.connectivity_extra_global_params)
model.reinitialise()
```
